# Skin microbiota secretomes modulate cutaneous innate immunity against *Borrelia burgdorferi* s.s

**DOI:** 10.1038/s41598-023-43566-0

**Published:** 2023-09-29

**Authors:** F. Baquer, B. Jaulhac, C. Barthel, M. Paz, J. Wolfgramm, A. Müller, N. Boulanger, A. Grillon

**Affiliations:** 1 Institut de Bactériologie, Fédération de Médecine Translationnelle de Strasbourg, University of Strasbourg, UR7290, ITI InnoVec, 3 Rue Koeberlé, 67000 Strasbourg, France; 2 Laboratory of Bacteriology, Strasbourg University Hospital, 67000 Strasbourg, France; 3 French National Reference Center for Borrelia, Strasbourg University Hospital, 67000 Strasbourg, France

**Keywords:** Infection, Bacterial host response

## Abstract

In Lyme borreliosis, the skin constitutes a major interface for the host, the bacteria and the tick. Skin immunity is provided by specialized immune cells but also by the resident cells: the keratinocytes and the fibroblasts. Discoveries on the role of the microbiome in the modulation of skin inflammation and immunity have reinforced the potential importance of the skin in vector-borne diseases. In this study, we analyzed in vitro the interaction of human primary keratinocytes and fibroblasts with *Borrelia burgdorferi* sensu stricto N40 in presence or absence of bacterial commensal supernatants. We aimed to highlight the role of resident skin cells and skin microbiome on the inflammation induced by *B. burgdorferi* s.s.. The secretomes of *Staphylococcus epidermidis*, *Corynebacterium striatum* and *Cutibacterium acnes* showed an overall increase in the expression of IL-8, CXCL1, MCP-1 and SOD-2 by fibroblasts, and of IL-8, CXCL1, MCP-1 and hBD-2 in the undifferentiated keratinocytes. Commensal bacteria showed a repressive effect on the expression of IL-8, CXCL1 and MCP-1 by differentiated keratinocytes. Besides the inflammatory effect observed in the presence of *Borrelia* on all cell types, the cutaneous microbiome appears to promote a rapid innate response of resident skin cells during the onset of *Borrelia* infection.

## Introduction

Lyme borreliosis is the first vector-borne disease in the temperate zone of the Northern Hemisphere^1^. It is caused by a spirochete belonging to the *Borrelia burgdorferi* sensu lato (*Borrelia* Lyme Group) and 23 species are currently attached to it^2^. The disease is transmitted by hard ticks *Ixodes* spp. during the blood meal^3^. In arthropod-borne diseases such as borreliosis, the skin of the vertebrate host plays a key role as the first interface encountered by pathogens^4^. There, arthropod saliva and pathogens are co-inoculated in the skin and interact with resident skin cells, keratinocytes (KCs), fibroblasts (FBs) and immune cells^5,6^. In a mouse model of Lyme borreliosis, skin has been showed to be a multiplication site before disseminating to target organs^7–9^. Multiple studies have highlighted the crucial importance of tick saliva, immune escape factors from the bacteria and the relationship between *Borrelia* and immune cells in the persistence of the pathogen in the skin^10–18^. FBs also produce chemokines and cytokines and other molecules in response to *Borrelia* (e.g. IL-8, CXCL-1, MCP-1, SOD-2, MMP-3)^19,20^.

More generally, resident skin cells are implicated in the immune response against pathogens. KCs, representing 90% of epidermal cells, express Pattern-Recognition-Receptors (PRRs) that detect pathogens, produce Anti-Microbial-Peptides (AMPs) as β-defensins (*e.g.*hBD-2) and cathelicidin^21^, as well as chemokines and cytokines (*e.g.*IL-8 and CXCL-1)^22^. In recent years, it clearly appears that the immunity of the skin is closely related to its microbiota. The interaction between the commensal flora and the skin has become an actively research field. Some studies point to the role of *Staphylococcus epidermidis* in stimulating the secretion of antimicrobial peptides by activating TLR2 via phenol-soluble modulines δ (PSMs)^23,24^. In addition, *S. epidermis* also play a role in the education and maintenance of immunity by the secretion of PSMε which is an agonist of the FPR2 receptor responsible for the secretion of IL-8 and chemotaxis phenol-soluble modulines^25^. Regarding *Cutibacterium acnes*, some strains are known to exacerbate immunity in the context of acne through activation of TLR2 and TLR4 pathways by various chemotactic factors^26^. The secretome of *C. striatum* remains mostly unknown and under-studied. Finally, the study of interactions between commensal microbiome and pathogenic species demonstrates complex interactions. They take place at the nutritional level with inhibition of metabolism^27^, by blocking quorum sensing^28^ and colonization^29^ or by direct destruction by bacteriocin secretion^24,30,31^. The skin can be divided into sebaceous, wet and dry zones, which constitute different ecological niches due to different environmental characteristics. These zones are dominated respectively by *Cutibacterium* spp., *Staphylococcus s*pp. and *Corynebacterium* spp.^32^.

In skin wounds, it is known that the release of pro-inflammatory chemokines is necessary for the initiation of repair^33^. However, the presence of normal skin flora helps to avoid an inflammatory response that is deleterious to the tissue. For example, lipoteichoic acid from *S. epidermidis* is described as an inhibitor of the TLR3 pro-inflammatory signaling pathway on KCs^34^, whereas it stimulates inflammation on other cell types (inflammasome activation). Basal layer KCs divide to regenerate the epidermis and FBs remodel the extracellular matrix. Macrophages attracted by the chemokine MCP-1 also help remodeling by eliminating matrix and polynuclear debris and maintain inflammation by phagocytosing bacteria. Finally, the polynuclear cells are attracted by the secretion of the chemokines IL-8 and CXCL1 and promote the restructuring of the epidermis by creating reticulated structures by the phenomenon of netosis^33^. The commensal flora therefore plays an important modulating role in the context of skin invasion.

During the bite of a hard tick, the hypostome and the two chelicerae of the arthropod penetrate the epidermis and are anchored into the dermis. This cutaneous injury develops during the course of a long-lasting blood meal of several days. Then during the infectious bite, tick saliva, pathogenic *Borreliae* and commensal skin bacteria tightly interact within the skin environment^35^. Since no study has explored the relationship between *Borrelia*, skin microbiota and resident skin cells, we therefore used an in vitro model of incubation and co-incubation of resident skin cells (KCs and FBs) by secretomes of bacteria representative of the main skin flora: *C. acnes*, *S. epidermidis*, *C. striatum*; in the presence or absence of *B.* *burgdorferi* s.s. strain N40.

## Results

### Interaction Undifferentiated keratinocytes-*Borrelia*-commensal bacteria

**Figure 1:**
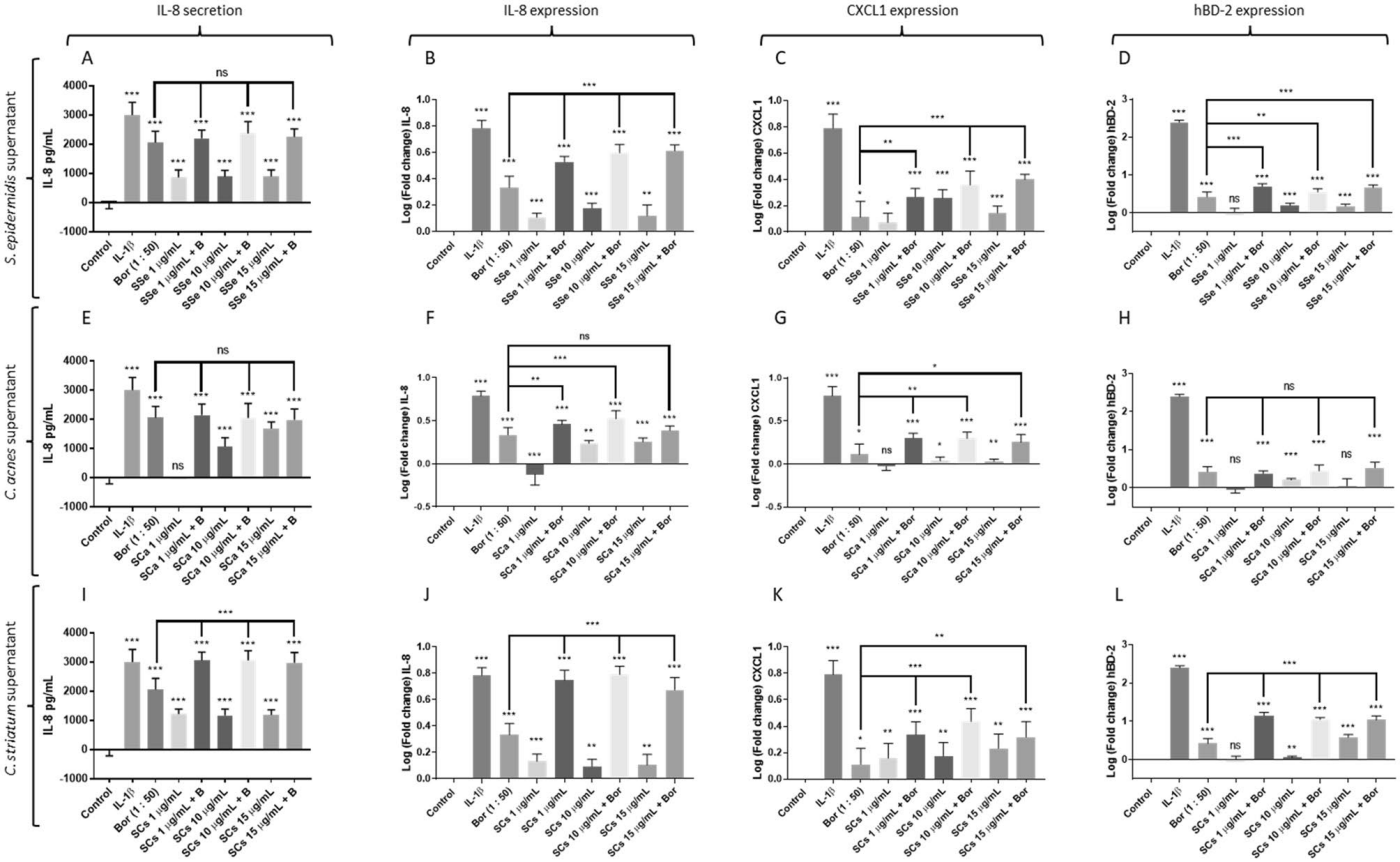
Measure of secreted IL-8 protein, fold change expression of IL-8, CXCL and hBD2 genes by undifferentiated human primary keratinocytes activated with/without Borrelia burgdorferi s.s N40 strain (Bor or B) and/or co-activated with (A, B, C, D) supernatant of Staphylococcus epidermidis (SSe) ; (E, F, G, H) supernatant of Cutibacterium acnes SCa; (I, J, K, L) supernatant of Corynebacterium striatum (SCs); (*** p ≤ 0.001, ** p ≤ 0.01, * p ≤ 0.05, ns = not significant).

*Borrelia burgdorferi s.s.* strain N40 alone (MOI 1:50) strongly induced IL-8 and hBD-2 gene expression (p < 0.0001) and moderately CXCL1 gene expression (p = 0.0173) of undifferentiated KCs (Fig. 1). Interestingly, supernatants of the different commensal bacteria weakly induced some of these inflammatory genes: *S. epidermidis* supernatant (SSe) and *C. striatum* supernatant (SCs) induced IL-8 (expression and secretion) and CXCL-1 whatever the concentration used (Fig. 1A,B,C,I,J,K). For hBD-2, a similar effect of induction was also observed, but only for the highest concentrations (10 and 15 μg/L) (Fig. 1D,L).

On the other hand, low concentration of *C. acnes* supernatant (SCa) (1 μg/L) did not trigger an inflammation of undifferentiated KCs (Fig. 1E,G,H); even a down regulation of IL-8 gene expression was observed (Fig. 1F). At higher concentrations (10 and 15 µg/mL), SCa incubations showed inducing effect on the IL-8 gene expression (Fig. 1E,F). A very weak inducing effect was also noticeable on the CXCL-1 and hBD-2 genes expression (10 µg/mL) (Fig. 1G,H).

Regarding co-incubations, the three commensal bacteria demonstrated synergistic effects with *Borrelia* N40 on IL-8 and CXCL-1 gene expressions by undifferentiated KCs (Fig. 1B,C,F,G,J,K), but the measure of IL-8 secretion by ELISA was only apparent for SCs, no matter the concentration used (Fig. 1I). Finally, a synergistic effect of commensal bacteria with *Borrelia* N40 strain on hBD-2 gene expression was also observed with SSe and SCs, but not for SCa (Fig. 1D,H,L).

### Differentiated keratinocytes-*Borrelia*-commensal bacteria

**Figure 2:**
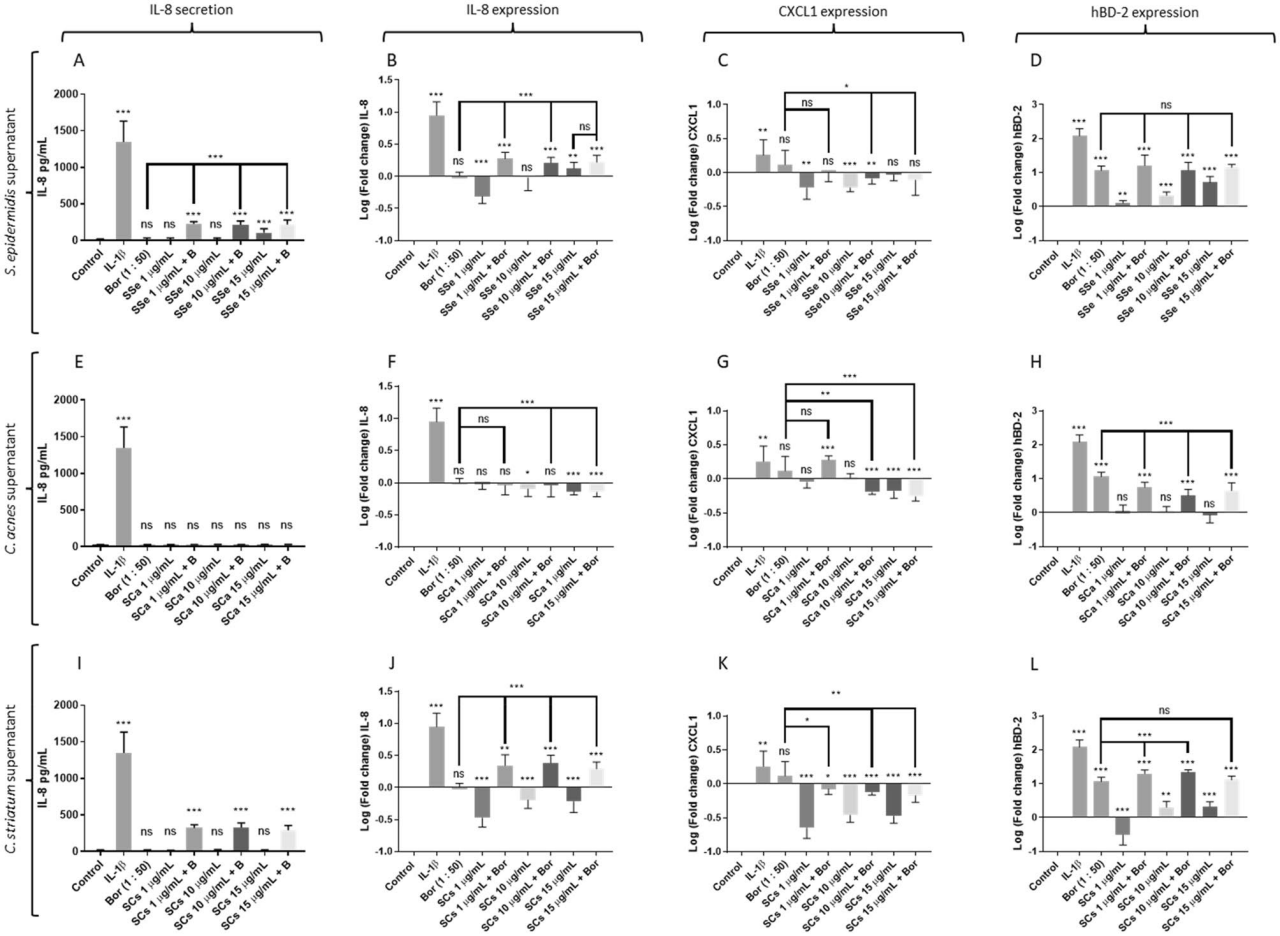
Measure of secreted IL-8 protein, fold change expression of IL-8, CXCL and hBD2 genes by differentiated human primary keratinocytes activated with/without Borrelia burgdorferi s.s N40 (Bor or B) and/or co-activated with (A, B, C, D) supernatant of Staphylococcus epidermidis (SSe) ; (E, F, G, H) supernatant of Cutibacterium acnes SCa; (I, J, K, L) supernatant of Corynebacterium striatum (SCs); (*** p ≤ 0.001, ** p ≤ 0.01, * p ≤ 0.05, ns = not significant).

Unlike undifferentiated KCs, *Borrelia* N40 strain alone (MOI 1:50) did not trigger IL-8 gene expression and secretion, nor CXCL-1 gene expression of differentiated KCs (Fig. 2A–C). An induction of hBD-2 gene expression was noticeable (p < 0.0001) (Fig. 2D).

Concerning SSe, a strong down regulation of IL-8 and CXCL-1 was observed with low and medium tested concentrations (Fig. 2B,C). This led to weak or absent IL-8 secretion (Fig. 2A). For hBD-2 gene expression, a dose dependent up-regulation was detected (Fig. 2D).

A similar effect, but more pronounced, was observed with SCs, with a strong down-regulation of IL-8 and CXCL-1 gene expression (Fig. 2I,J,K). Even hBD-2 gene expression was repressed by low concentration of SCs, followed by a weak induction at higher concentrations (Fig. 2L). About SCa, a down-regulation of IL-8 expression was observed with medium and high tested concentration (p = 0.0308, p < 0.0001) without modification of IL-8 secretion (Fig. 2E,F) and a repressive effect on CXCL1 was detected for high tested concentration (Fig. 2G). However, no noticeable effect on hBD-2 expression was observed with SCa (Fig. 2H).

Regarding co-incubations, surprisingly, a synergistic effect between *Borrelia* and SSe or SCs was observed on IL-8 gene expression and secretion (Fig. 2A,B,I,J). This effect was not noticeable with SCa (Fig. 2E,F). Effect of co-incubations on CXCL-1 gene expression showed a mild to strong down regulation with the three supernatants. This effect was higher with SCa and SCs than with SSe (Fig. 2C,G,K). Finally, SSe combined with *Borrelia* N40 did not alter hBD-2 expression compared to *Borrelia* N40 alone (Fig. 2D), SCa decreased hBD-2 induction by *Borrelia* N40 (Fig. 2H) while SCs increased hBD-2 induction only at concentrations of 1 and 10 µg/mL of *Borrelia* N40 (Fig. 2L).

### Fibroblasts*-Borrelia*-commensal bacteria

**Figure 3:**
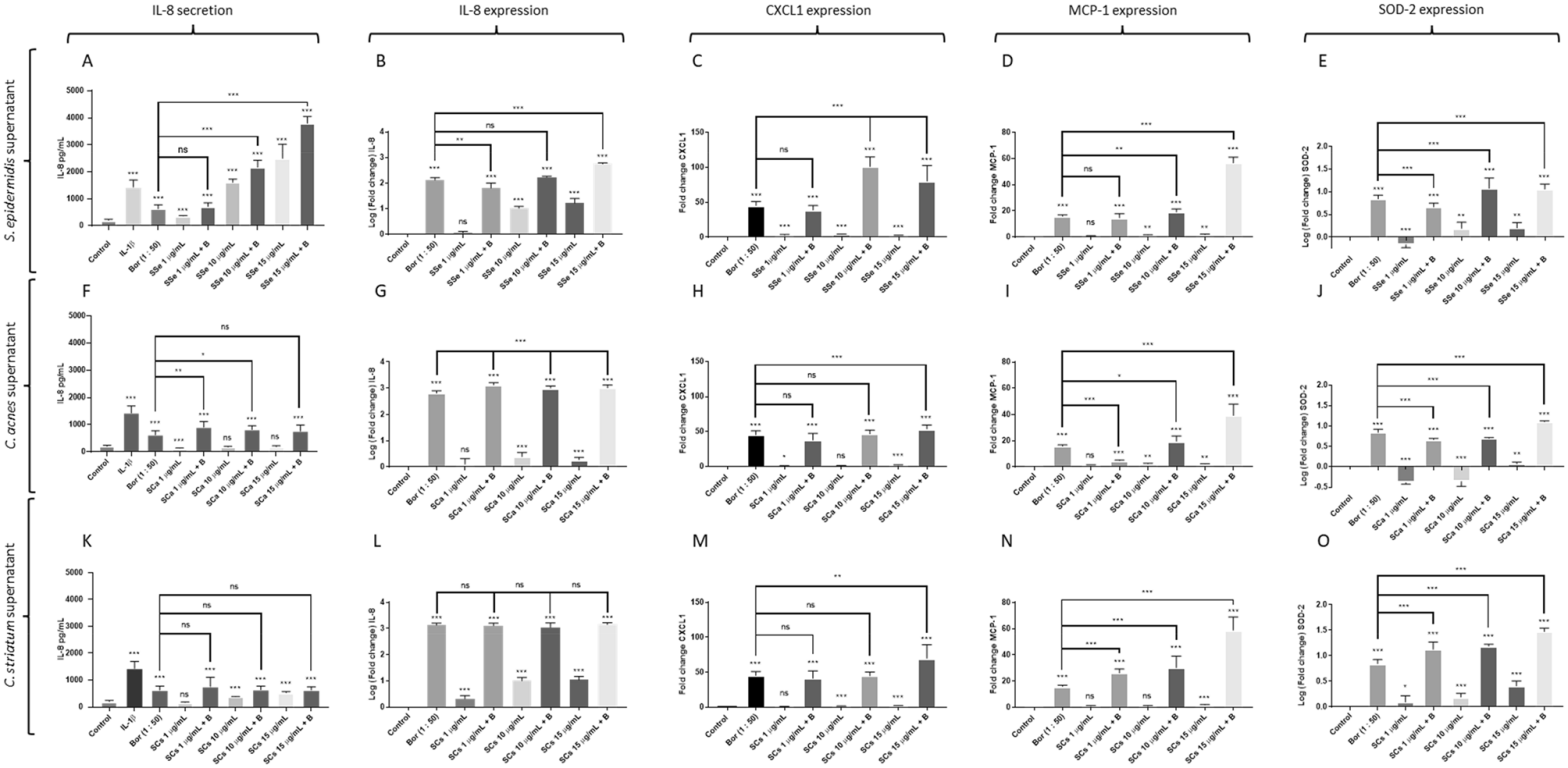
Measure of secreted IL-8 protein, fold change expression of IL-8, CXCL and hBD2 genes by human primary fibroblasts activated with/without Borrelia burgdorferi s.s N40 (Bor or B) and/or co-activated with (A, B, C, D) supernatant of Staphylococcus epidermidis (SSe) ; (E, F, G, H) supernatant of Cutibacterium acnes SCa; (I, J, K, L) supernatant of Corynebacterium striatum (SCs); (*** p ≤ 0.001, ** p ≤ 0.01, * p ≤ 0.05, ns = not significant).

As expected, *Borrelia* N40 strain induced IL-8 secretion and upregulation of IL-8, CXCL-1, MCP-1 and SOD-2 gene expression (Fig. 3). The only negative result was observed with MMP-3, where *Borrelia* N40 showed no effect on this gene expression (data not shown).

The three commensal bacteria supernatants alone induced an upregulation of IL-8 gene (Fig. 3B,G,L). This upregulation triggered an IL-8 secretion only by SSe and SCs, while little to no effect was observed with SCa (Fig. 3A,F,K). A small dose dependent effect on CXCL-1 and MCP-1 gene expression was also observed (Fig. 3C,D,H,I,M,N). Regarding SOD-2, a down-regulation was noticeable with SCa and SSe at the lowest concentration (1 µg/mL) (Fig. 3E,J). At higher concentrations, this effect continued to be observed with SCa, while SSe and SCs induced a weak positive response, weaker than *Borrelia* N40 alone (Fig. 3E,J,O).

Co-incubations of *Borrelia* N40 with the three supernatants showed different profiles. SSe with *Borrelia* N40 showed almost systematically a dose dependent synergistic effect on cytokines and chemokines tested (Fig. 3A–E). With SCs or SCa and *Borrelia*, this synergistic effect was only visible for MCP-1 and SOD-2 gene expression (Fig. 3N, O) while no remarkable effect was observed on IL-8 and CXCL-1 gene expression (Fig. 3L, M). Only high concentrations (15 µg/mL) of SCa or SCs triggered a CXCL-1 upregulation (Fig. 3H, M).

## Discussion

The skin microbiome became a key element in different skin disorders^36^. However, its role in vector-borne disease has been poorly investigated. One early study reported that germfree mice are more susceptible to *Leishmania major*, a parasite transmitted by the sandfly^37^. More recently, in C57BL/6 germfree mouse, parasite multiplication was higher than in normal NMRI mice due to a lower number of CD4 + T cells producing cytokines interferon-γ (IFN-γ) controlling the infection. When the authors co-inoculated *S. epidermidis* with *Leishmania* in the latter mice, IFN-γ secreted by T cells was produced again and it rescued protective immunity against the parasitic infection^38^.

The present work focuses on the effect of different commensal bacterial secretomes on the inflammation induced by *Borrelia* co-incubated with resident skin cells in different culture models. We used the secretomes since the bacterial wall components were shown to produce a systematic death of FBs and KCs (data not shown). Indeed, the lipoteichoic acids contained in the wall of certain species of Gram-positive bacteria can cause a significant toxicity^39–41^. In this context, the bicompartmental culture model or the use of the supernatant are the best alternatives. The use of the pooled secretome from several strains limits the biases related to the differences between strains and increases the reproducibility of the results. However, the use of a secretome can raise the question of the actual concentration of the bacterial secretome on the skin surface, but our model is designed to explore the effects of the secretome components to further identify them and characterize the pathways involved.

Our experimental model explored the role of two major resident skin cells, the KCs of the epidermis and the FBs of the dermis.

*KCs are major protagonists*; they make up the epidermis and are the first to be activated during skin invasion and play a sentinel role^42^. They efficiently detect PAMPs and produce cytokines and PAMs. KCs differentiate as they progress to the outer layers of the skin. Differentiated KCs are in close contact with the skin microbiota, but they are less easily activated by stimuli due to the progressive loss of their nuclei as shown in this study. Bacterial secretomes have an overall repressive effect on differentiated KCs. The *S. epidermidis* secretome is an inducer of hBD-2, as described in the literature^23^. Co-incubation with *Borrelia* widely increases the expression of the studied genes.

The low level of incubation of differentiated KCs in the presence of *Borrelia* can be explained by the low reactivity of these cells. In contrast, commensal bacteria supernatants induced a weak inflammation on undifferentiated KCs. Co-incubations by *Borrelia* revealed a synergistic effect for SSe and SCa and a likely additional effect with SCs. Incubation of KCs by the commensal microbiota probably reflects the intrinsic role of control of innate immunity by the skin flora. These results are in accordance with the phenomenon of tolerance between the epidermis and the cutaneous microbiota present on the surface of the skin^43^ and with the role of education and potentiation of innate immunity.

*Concerning the FBs*, they are a target of choice since during the tick bite, the hypostome and the two chelicerae are embedded in the dermis and then, the tick saliva and *Borrelia* come into direct contact with the FBS. They are known to play an amplifying role in the activation of innate immunity and are capable of producing a wide variety of cytokines and chemokines^44,45^. SSe and SCs supernatants had an inducing effect on IL-8 secretion and transcription, the effect was much less pronounced with SCa. In addition, all three secretomes had a moderate inducing effect on the expression of the CXCL1 and MCP-1 chemokines. The modulation of SOD-2 expression was more complex, with upregulation at high concentration and downregulation at low concentration for SSe and SCa. SOD-2 expression usually increases in an inflammatory context; therefore, this modulation may reflect the absence of deleterious inflammation caused by the microbiota. As a co-incubation, commensal secretomes with *Borrelia* brings, presented a strong synergistic effect on IL-8 transcription and secretion in the presence of SSe while this effect seemed to be additional with SCa or SCs. All other genes were affected by a synergistic or additional effect in the presence of secretomes. No modulation of MMP-3 expression was observed by exposure to the secretome or *Borrelia* (data not shown). This may indicate that the detection of *Borrelia* by FBs did not induce early remodeling of the extracellular matrix facilitating pathogen dissemination.

Physiologically, the cutaneous microbiota mainly interacts with the KCs of the superficial layers in a mutualistic way. Our results underline the pro-inflammatory power of skin flora on FBs and undifferentiated KCs during a skin injury such as a tick bite.

Co-incubation of skin resident cells shows that skin flora potentiates inflammatory signaling in the presence of *Borrelia*, already at the dose of 1 µg/ml. This effect is particularly marked for FBs. The discovery of synergistic and sometimes additive effects suggests the activation of different inflammation pathways by several molecules. The PRRs most often involved in bacterial recognition are the TLRs, which are widely expressed by KCs and FBs^46^. The most involved receptor is probably TLR-2 which recognizes lipoteichoic acids but also the lipoproteins OspA and OspC of *Borrelia*^47^. This receptor is responsible for the activation of the MAPK and NF-κb pathways^46^. These pathways control the upregulation of IL-8, CXCL1 and MCP-1^48^. TLR-4 is unlikely to be activated by the three skin bacteria tested here which are all Gram (+) or *Borrelia*, which do not possess lipopolysaccharide, explaining the lack of TNF-α induction (data not shown). Identification of the molecules of the different secretomes involved in the inflammation deserve to be done.

The co-incubation model shows that the skin microbiota potentiates the early innate response of skin resident cells to *Borrelia*. The importance of this incubation on the clearance of *Borrelia* by the immune system remains unclear in this in vitro model. Moreover, the intensity of incubation probably varies according to the strain of *B. burgdorferi* s.s.. It has been described that certain strains of the RST1 group induce greater production of interleukins and chemokines by macrophages and peripheral blood mononuclear cells than other genotypes^49^. New models including professional immune cells and tick saliva are needed to improve our understanding of their interactions. Indeed, tick saliva is well-known for its immunosuppressive effect and it likely affects the early inflammation during the tick bite process^35^. In addition, commensal bacteria modulate differently the skin inflammatory response. The anatomical diversity of the distribution of skin microbiota populations could possibly also influence the efficiency of early transmission depending on the bite site.

This in vitro model has its limitations since it does not take into account the impact of other immune cells, present in the skin. It focuses on the innate and early immunomodulatory effects of the resident cells of the dermis and epidermis. The use of animal models with controlled skin microbiota could allow a more detailed understanding of its role in the process of *Borrelia* transmission by *Ixodes* tick.

## Material and method

### Spirochete strain

*B. burgdorferi* s.s. strain N40 (North American strain isolated from *I. scapularis*) was used at passage 8, cultured in BSK-H medium (Sigma, Saint Quentin Fallavier, France) at 33 °C with 5% CO_2_, and the pellet washed twice before the assays to discard BSK-H medium remnants and replace it by KGM-gold medium (Lonza, Basel, Switzerland) for KC experiments or FGM medium (Promocell, Heidelberg, Germany) for FB experiments. Bacterial viability was previously tested in KGM and FGM.

### Bacterial cultures and secretome preparation

A total of 5 distinct strains for each commensal bacteria species isolated from human healthy skin samples at the bacteriology laboratory of Strasbourg University Hospital were used for the study. Samples were taken for routine ecological screening purposes, and each adult patient was checked for the absence of dermatological diseases. Detection of plasmids was not performed. All strains have been cultured in liquid Muller Hinton medium, 37 °C for 24h in aerobic conditions for *S. epidermidis* and *C. striatum* and in anaerobic environment for *C. acnes* using GenBag anaerobic (Biomerieux, Marcy l’Etoile, France). Sterile supernatants of *S. epidermidis* (SSe), *C. acnes* (SCa) and *C. striatum* (SCs) were obtained after filtration (Nalgène 0.20 µm filters—Thermofisher). Sterile secretomes of each species were pooled and protein concentration was measured on ADVIA 2400 (Siemens). Strain purity and supernatant sterility were controlled by agar plate culture.

### Cell cultures and incubations

Human Primary dermal FBs (NHDF, Promocell, Heidelberg, Germany) were maintained in FGM2 medium (Promocell, Heidelberg, Germany). Primary foreskin human KCs were obtained from pediatric surgery department of Strasbourg University Hospital (ethics committee registration number: Pri2011-hus-5093). KCs were maintained in KGM-Gold™ media supplemented with Bullekit™ supplements (Promocell, Heidelberg, Germany).

To stimulate the cells, FBs and KCs were used at passage 3 to 5 and seeded at 7.5 × 10^4^ per well in a 24-well plate. For FBs incubation, at confluence and one day before incubation, FGM2 medium was replaced by FGM medium without foetal calf serum. For KCs incubation, cells were used at 80% confluence. To mimic normal skin, undifferentiated KCs and differentiated KCs were used. To obtain differentiated KCs, CaCl2 at 1.5 mM was added on the 80% confluence KCs layer 24 h before experiments. FBs and KCs were stimulated by the different secretomes of SSe, SCa and SCs alone, at different concentrations (1, 10 and 15 μg/L), diluted in the appropriate culture media (FGM or KBM-gold), for 24 h. *B.* *burgdorferi* s.s. strain N40 alone at multiplicity of infection (MOI) of 50:1 for 24 h or co-stimulated by *B. burgdorferi* s.s. N40 and SSe, SCa and SCs at each concentration for 24 h. For KCs and FBs, IL-1β was used as a positive control for secretion and/or expression of IL-8, CXCL-1 and hBD-2 (Table 1), unstimulated cell cultures were used as negative control.

**Table 1:** Cell culture and incubation workflow (MOI 50:1 = 50 bacteria per cell).

Cells	Expansion medium	Incubation medium	Bacterial supernatant	*B*. *burgdorferi* s.s. N40	Positive control	Negative control
Fibroblasts (FBs)	FGM2	FGM	*S. epidermidis* 1, 10 or 15 µg/L	None or MOI 50:1	IL-1β	FGM
			*C. acnes* 1, 10 or 15 µg/L			
			*C. stria*tum 1, 10 or 15 µg/L			
			None	MOI 50:1		
Undifferentiated keratinocytes (KCs)	KGM		*S. epidermidis* 1, 10 or 15 µg/L	None or MOI 50:1		KGM
			*C. acnes* 1, 10 or 15 µg/L			
			*C. striatum* 1, 10 or 15 µg/L			
			None	MOI 50:1		
Differentiated keratinocytes (KCs)			*S. epidermidis* 1, 10 or 15 µg/L	None or MOI 50:1		
			*C. acnes* 1, 10 or 15 µg/L			
			*C. striatum* 1, 10 or 15 µg/L			
			None	MOI 50:1		

### IL-8 and TNFα ELISA

IL-8 secretion levels were measured in culture supernatants of unstimulated and *Borrelia*-stimulated cells by ELISA. The protocol was based on sandwich techniques (DuoSet® Human CXCL8/IL-8, Cat# DY208), as described by the manufacturer (R&D systems, Lille, France). Results are representative of three independent experiments.

### RNA extraction and semi-quantitative real time PCR

After removal of the supernatant, FBs were immediately lysed using RNA Lysis buffer (Zymo Research, Irvine, California, United States). Duplicate wells were pooled and RNA extract were stored at – 80 °C. Then, total RNA were extracted using Quick-RNA Miniprep kit (Zymo Research, Irvine, California, United States) according to the manufacturer’s protocol.

Total RNA (500 µg) was reverse transcribed with the Superscript II first-strand synthesis system (Invitrogen, Cergy-Pontoise, France). Semiquantitative reverse transcription PCR (QRT-PCR) was done on an ABI Prism 7000 (Applied Biosystems, Courtaboeuf,France) with specific primers and Power Sybrgreen reagent (Applied Biosystems, Courtaboeuf,France). The transcriptional expression of IL-8, CXCL1, MCP-1, SOD-2 and MMP-3 genes were assessed by qRT-PCR on FBs while the targets for KCs were IL-8, CXCL1 and hBD-2 transcripts. Expression levels of all transcripts studied were normalized to β-actin housekeeping gene level and the relative changes in gene expression were compared with those of untreated cells using the 2^−ΔΔCt^ method.

### Statistics

For ELISA and QRT-PCR, each experiment of cell incubation with bacteria and secretome was carried out at least three times in independent experiments and all quantifications were performed in triplicate. Results are presented as means ± standard deviations (SDs) and were analyzed by Welsh’s test. Differences in values were considered significant at p < 0.05. For ELISAs, the data were the means ± SDs of triplicate values and were representative of three independent experiments. For qRT-PCR, the values were normalized to the negative control (medium alone) for each experiment and shown as the fold change or Log Fold change of the control’s value. The means ± SDs of triplicate values were compared between stimulated and unstimulated cells and are representative of three independent experiments. Gaussian distribution of every condition has been assessed by Shapiro’s test with alpha risk < 5%.

### Supplementary Information

Supplementary Information 1.

Supplementary Information 2.

Supplementary Information 3.

## Data Availability

The datasets analysed during the current study are available in supplementary data. The datasets not shown are available from the corresponding author on reasonable request.

## References

[1] Stanek G, Wormser GP, Gray J, Strle F (2012). Lyme borreliosis. Lancet Lond. Engl..

[2] Trevisan G, Cinco M, Trevisini S, di Meo N, Chersi K, Ruscio M (2021). Borreliae Part 1: Borrelia lyme group and echidna-reptile group. Biology.

[3] Radolf JD, Caimano MJ, Stevenson B, Hu LT (2012). Of ticks, mice and men: Understanding the dual-host lifestyle of Lyme disease spirochaetes. Nat. Rev. Microbiol..

[4] Bernard Q, Jaulhac B, Boulanger N (2014). Smuggling across the border: How arthropod-borne pathogens evade and exploit the host defense system of the skin. J. Invest. Dermatol..

[5] Bernard Q, Gallo RL, Jaulhac B, Nakatsuji T, Luft B, Yang X (2016). Ixodes tick saliva suppresses the keratinocyte cytokine response to TLR2/TLR3 ligands during early exposure to Lyme borreliosis. Exp. Dermatol..

[6] Boeuf A, Schnell G, Bernard Q, Kern A, Westermann B, Ehret-Sabatier L (2019). Dissociating effect of salivary gland extract from *Ixodes*
*ricinus* on human fibroblasts: Potential impact on Borrelia transmission. Ticks Tick-Borne Dis..

[7] Kern A, Collin E, Barthel C, Michel C, Jaulhac B, Boulanger N (2011). Tick saliva represses innate immunity and cutaneous inflammation in a murine model of lyme disease. Vector Borne Zoonotic Dis. Larchmt NY.

[8] Bernard Q, Wang Z, Di Nardo A, Boulanger N (2017). Interaction of primary mast cells with *Borrelia*
*burgdorferi* (sensu stricto): Role in transmission and dissemination in C57BL/6 mice. Parasit. Vectors.

[9] Horká H, Cerná-Kýcková K, Skallová A, Kopecký J (2009). Tick saliva affects both proliferation and distribution of *Borrelia*
*burgdorferi* spirochetes in mouse organs and increases transmission of spirochetes to ticks. Int. J. Med. Microbiol. IJMM.

[10] Önder Ö, Humphrey PT, McOmber B, Korobova F, Francella N, Greenbaum DC (2012). OspC is potent plasminogen receptor on surface of *Borrelia*
*burgdorferi*. J. Biol. Chem..

[11] Liang FT, Yan J, Mbow ML, Sviat SL, Gilmore RD, Mamula M (2004). *Borrelia*
*burgdorferi* changes its surface antigenic expression in response to host immune responses. Infect. Immun..

[12] Ribeiro JM, Makoul GT, Levine J, Robinson DR, Spielman A (1985). Antihemostatic, antiinflammatory, and immunosuppressive properties of the saliva of a tick *Ixodes*
*dammini*. J Exp Med..

[13] Ribeiro JM, Spielman A (1986). *Ixodes*
*dammini*: salivary anaphylatoxin inactivating activity. Exp. Parasitol..

[14] Juncadella IJ, Anguita J (2009). The immunosuppresive tick salivary protein, Salp15. Adv. Exp. Med. Biol..

[15] Hannier S, Liversidge J, Sternberg JM, Bowman AS (2004). Characterization of the B-cell inhibitory protein factor in *Ixodes*
*ricinu*s tick saliva: a potential role in enhanced *Borrelia*
*burgdoferi* transmission. Immunology.

[16] Sajiki Y, Konnai S, Ochi A, Okagawa T, Githaka N, Isezaki M (2020). Immunosuppressive effects of sialostatin L1 and L2 isolated from the taiga tick *Ixodes*
*persulcatus* Schulze. Ticks Tick-Borne Dis..

[17] Marchal CMP, Luft BJ, Yang X, Sibilia J, Jaulhac B, Boulanger NM (2009). Defensin is suppressed by tick salivary gland extract during the in vitro interaction of resident skin cells with *Borrelia*
*burgdorferi*. J. Invest. Dermatol..

[18] Marchal C, Schramm F, Kern A, Luft BJ, Yang X, Schuijt TJ (2011). Antialarmin effect of tick saliva during the transmission of Lyme disease. Infect. Immun..

[19] Schramm F, Kern A, Barthel C, Nadaud S, Meyer N, Jaulhac B (2012). Microarray analyses of inflammation response of human dermal fibroblasts to different strains of *Borrelia*
*burgdorferi* sensu stricto. PloS One..

[20] Zhao Z, Fleming R, McCloud B, Klempner MS (2007). CD14 mediates cross talk between mononuclear cells and fibroblasts for upregulation of matrix metalloproteinase 9 by *Borrelia*
*burgdorferi*. Infect. Immun..

[21] Ong PY, Ohtake T, Brandt C, Strickland I, Boguniewicz M, Ganz T (2002). Endogenous antimicrobial peptides and skin infections in atopic dermatitis. N. Engl. J. Med..

[22] Albanesi C, Scarponi C, Giustizieri ML, Girolomoni G (2005). Keratinocytes in inflammatory skin diseases. Curr. Drug Targets Inflamm. Allergy..

[23] Lai Y, Cogen AL, Radek KA, Park HJ, Macleod DT, Leichtle A (2010). Activation of TLR2 by a small molecule produced by *Staphylococcus epidermidis* increases antimicrobial defense against bacterial skin infections. J. Invest. Dermatol..

[24] Cogen AL, Yamasaki K, Muto J, Sanchez KM, Crotty Alexander L, Tanios J (2010). *Staphylococcus epidermidis* antimicrobial delta-toxin (phenol-soluble modulin-gamma) cooperates with host antimicrobial peptides to kill group A Streptococcus. PloS One..

[25] Li S, Huang H, Rao X, Chen W, Wang Z, Hu X (2014). Phenol-soluble modulins: novel virulence-associated peptides of staphylococci. Future Microbiol..

[26] Nagy I, Pivarcsi A, Koreck A, Széll M, Urbán E, Kemény L (2005). Distinct strains of *Propionibacterium acnes* induce selective human beta-defensin-2 and interleukin-8 expression in human keratinocytes through toll-like receptors. J. Invest. Dermatol..

[27] Wang Y, Kuo S, Shu M, Yu J, Huang S, Dai A (2014). *Staphylococcus epidermidis* in the human skin microbiome mediates fermentation to inhibit the growth of Propionibacterium acnes: Implications of probiotics in acne vulgaris. Appl. Microbiol. Biotechnol..

[28] Paharik AE, Parlet CP, Chung N, Todd DA, Rodriguez EI, Van Dyke MJ (2017). Coagulase-negative staphylococcal strain prevents *Staphylococcus aureus* colonization and skin infection by blocking quorum sensing. Cell Host Microbe..

[29] Krauss S, Zipperer A, Wirtz S, Saur J, Konnerth MC, Heilbronner S (2020). Secretion of and self-resistance to the novel fibupeptide antimicrobial lugdunin by distinct ABC transporters in *Staphylococcus **lugdunensis*. Antimicrob. Agents Chemother..

[30] Bastos MCF, Ceotto H, Coelho MLV, Nascimento JS (2009). Staphylococcal antimicrobial peptides: Relevant properties and potential biotechnological applications. Curr. Pharm. Biotechnol..

[31] Claesen J, Spagnolo JB, Ramos SF, Kurita KL, Byrd AL, Aksenov AA (2020). A *Cutibacterium** acnes* antibiotic modulates human skin microbiota composition in hair follicles. Sci Transl Med..

[32] Sanford JA, Gallo RL (2013). Functions of the skin microbiota in health and disease. Semin. Immunol..

[33] Rodrigues M, Kosaric N, Bonham CA, Gurtner GC (2019). Wound healing: A cellular perspective. Physiol. Rev..

[34] Lai Y, Di Nardo A, Nakatsuji T, Leichtle A, Yang Y, Cogen AL (2009). Commensal bacteria regulate Toll-like receptor 3-dependent inflammation after skin injury. Nat. Med..

[35] Boulanger N, Wikel S (2021). Induced transient immune tolerance in ticks and vertebrate host: A keystone of tick-borne diseases?. Front Immunol..

[36] Chen Y, Knight R, Gallo RL (2023). Evolving approaches to profiling the microbiome in skin disease. Front. Immunol..

[37] de Oliveira MR, Tafuri WL, Nicoli JR, Vieira EC, Melo MN, Vieira LQ (1999). Influence of microbiota in experimental cutaneous leishmaniasis in Swiss mice. Rev. Inst. Med. Trop. Sao Paulo.

[38] Naik S, Bouladoux N, Wilhelm C, Molloy MJ, Salcedo R, Kastenmuller W (2012). Compartmentalized control of skin immunity by resident commensals. Science..

[39] Leon O, Panos C (1983). Cytotoxicity and inhibition of normal collagen synthesis in mouse fibroblasts by lipoteichoic acid from *Streptococcus pyogenes* type 12. Infect. Immun..

[40] Morath S, Stadelmaier A, Geyer A, Schmidt RR, Hartung T (2002). Synthetic lipoteichoic acid from *Staphylococcus aureus* is a potent stimulus of cytokine release. J. Exp. Med..

[41] Goldschmidt JC, Panos C (1984). Teichoic acids of *Streptococcus **agalactiae*: chemistry, cytotoxicity, and effect on bacterial adherence to human cells in tissue culture. Infect Immun..

[42] Nestle FO, Di Meglio P, Qin JZ, Nickoloff BJ (2009). Skin immune sentinels in health and disease. Nat. Rev. Immunol..

[43] Gallo RL, Nakatsuji T (2011). Microbial symbiosis with the innate immune defense system of the skin. J. Invest. Dermatol..

[44] Kendall RT, Feghali-Bostwick CA (2014). Fibroblasts in fibrosis: novel roles and mediators. Front. Pharmacol..

[45] Buechler MB, Turley SJ (2018). A short field guide to fibroblast function in immunity. Semin. Immunol..

[46] Miller LS (2008). Toll-like receptors in skin. Adv. Dermatol..

[47] Kawai T, Akira S (2007). TLR signaling. Semin. Immunol..

[48] Burke SJ, Lu D, Sparer TE, Masi T, Goff MR, Karlstad MD (2014). NF-κB and STAT1 control CXCL1 and CXCL2 gene transcription. Am. J. Physiol. Endocrinol. Metab..

[49] Strle K, Jones KL, Drouin EE, Li X, Steere AC (2011). *Borrelia*
*burgdorferi* RST1 (OspC type A) genotype is associated with greater inflammation and more severe Lyme disease. Am. J. Pathol..

